# Comprehensive social determinants of health and brain health in midlife

**DOI:** 10.1016/j.tjpad.2026.100652

**Published:** 2026-08-10

**Authors:** Christina S. Dintica, Julia Cheunkarndee, R. Nick Bryan, Lenore J. Launer, Kristine Yaffe

**Affiliations:** a University of California, San Francisco, San Francisco, CA, USA; b Northern California Institute for Research and Education, San Francisco, CA, USA; c Department of Radiology, University of Pennsylvania, Philadelphia, PA, USA; d Laboratory of Epidemiology and Population Sciences, National Institute on Aging Baltimore, MD, USA

**Keywords:** Social determinants of health, Cognitive decline, Brain aging, White matter hyperintensities, Midlife

## Abstract

**Background:**

Social determinants of health (SDOH) are increasingly recognized as important drivers of cognitive outcomes. However, most existing evidence focuses on individual SDOH components and older populations.

**Objectives:**

To develop a comprehensive SDOH index and examine its association with subsequent changes in cognitive function and structural brain measures in midlife.

**Design:**

Prospective cohort study with repeated measures of cognition and brain imaging.

**Setting:**

Community-based cohort from the Coronary Artery Risk Development in Young Adults (CARDIA) study.

**Participants:**

A total of 3488 participants with SDOH data in early midlife (mean age 40.0 ± 3.6 years); 645 participants had repeated brain magnetic resonance imaging (MRI) data.

**Measurements:**

A weighted aggregate SDOH index was constructed from 12 items across 5 domains: economic stability, community and social context, education, neighborhood and built environment, and health care access. Cognitive function was assessed using the Digit Symbol Substitution Test (DSST), Stroop Test, and Rey Auditory Verbal Learning Test (RAVLT). Brain MRI outcomes included white matter hyperintensities (WMHs) and total gray matter (GM) volume. Mixed linear regression models examined associations between SDOH quartiles and longitudinal cognitive and MRI outcomes, adjusting for demographics, vascular risk factors, depression, and intracranial volume (for MRI).

**Results:**

At baseline, participants in the most disadvantaged SDOH quartile performed worse across all cognitive tests compared with the least disadvantaged quartile (*p* < 0.001). Over time, the most disadvantaged quartile showed steeper decline in DSST performance (adj. mean change: −0.72, 95% CI: −0.93 to −0.52 vs. −0.55, 95% CI: −0.76 to −0.34, *p* = 0.013), greater WMH accumulation (ratio: 1.07, 95% CI: 1.05 to 1.09 vs. 1.04, 95% CI: 1.03 to 1.05, *p* = 0.007), and steeper decline in total GM volume (−2.02 cm³, 95% CI: −2.39 to −1.65 vs. −1.46 cm³, 95% CI: −1.71 to −1.20, *p* = 0.011) per 5-year interval compared to the least disadvantaged quartile.

**Conclusions:**

Greater social disadvantage in midlife is associated with worse baseline cognition and accelerated decline in cognitive function and brain integrity. These findings highlight the importance of SDOH as key determinants of brain health in midlife and suggest that strategies to mitigate social disadvantage may help preserve cognitive and brain health.

## 1 Introduction

Social determinants of health (SDOH) encompass the economic, social, and environmental conditions that shape health outcomes across the life course. These factors—such as socioeconomic status (SES), education, employment, social support, and access to healthcare—have been increasingly recognized as key contributors to cognitive and brain health [1,2]. While the influence of adverse SDOH on cognitive outcomes in late life is well established, much less is known about their effects on brain health during midlife, a period when neurobiological changes linked to aging and dementia begin to emerge [3,4].

Prior research on SDOH and cognitive aging has primarily focused on individual factors, such as low SES, financial instability, or social isolation, and their associations with dementia risk and cognitive decline. For example, studies have demonstrated that lower SES is linked to reduced cortical thickness, smaller hippocampal volume, and increased white matter lesions [5,6], Similarly, chronic exposure to financial instability has been associated with accelerated cognitive decline and poorer brain integrity in midlife, suggesting that economic hardship may have long-term neurobiological consequences [7]. Social engagement and support also play a critical role, with social isolation and limited social networks being linked to worse cognitive outcomes, independent of economic status [8,9]. However, studies examining the cumulative impact of multiple SDOH factors on brain health remain limited, and existing composite indices often focus narrowly on economic indicators while overlooking other critical domains such as social and environmental conditions.

Moreover, midlife represents a particularly important but understudied window for examining the effects of cumulative social disadvantage on brain aging. Neuropathological changes associated with dementia and cognitive decline often begin decades before clinical symptoms appear, making midlife a critical period for identifying early social risk factors [10,11]. Exposure to chronic stressors during midlife—such as financial strain, neighborhood disadvantage, and social isolation—has been associated with structural brain alterations, increased inflammation, and dysregulated stress responses, all of which may accelerate cognitive aging [12,13]. Despite this, research on the role of SDOH in midlife brain health remains scarce, with most studies focusing on older adults or relying on individual measures rather than comprehensive, multidimensional indices.

To address these gaps, we leverage longitudinal data from the Coronary Artery Risk Development in Young Adults (CARDIA) study to develop a cumulative SDOH index encompassing multiple domains outlined by the Healthy People 2030 framework: economic stability, education, neighborhood and physical environment, community and social context, and healthcare access and quality [14]. Using this index, we aim to evaluate how cumulative exposure to adverse SDOH in early midlife relates to subsequent changes in brain structure and cognitive performance later in midlife. By adopting a multidimensional approach, this study provides a more comprehensive understanding of how social disadvantage shapes brain health trajectories during midlife, offering critical insights for early intervention and dementia prevention.

## 2 Methods

### 2.1 Study population

The Coronary Artery Risk Development in Young Adults (CARDIA) study is a prospective cohort study investigating the development of and risk factors for cardiovascular disease [15]. Briefly, starting in 1985, 5115 Black and White adults between 18 and 30 years of age were recruited from population-based samples of 4 US cities (Birmingham, AL; Chicago, IL; Minneapolis, MN; and Oakland, CA). Within each center, recruitment was balanced by sex, race, age, and educational level. Participants completed follow-up examinations every 2 to 5 years for 30 years: 1987 to 1988 (Year 2), 1990 to 1991 (Year 5), 1992 to 1993 (Year 7), 1995 to 1996 (Year 10), 2000 to 2001 (Year 15), 2005 to 2006 (Year 20), 2010 to 2011 (Year 25), 2015–2016 (Year 30), and Year 35 (2020–2022).

At each examination, participants provided written informed consent, and study protocols were approved by institutional review boards at each study site and the CARDIA Coordinating Center. Further details regarding the design and recruitment of CARDIA have been previously reported [15].

### 2.2 SDOH index

As a summary of social burden, we created an aggregate SDOH index based on the framework described by the Healthy People 2030 initiative [14], which categorizes SDOH into 5 domains: economic stability, neighborhood and physical environment, education, community and social context, and health care access and quality. In CARDIA, economic stability measures included income, house tenure, and financial strain. Education was assessed with formal educational attainment (years). Community and social context included measures on social support, chronic stress, neighborhood cohesion, and discrimination. Health care access and quality included information about having a usual source of care, health insurance, and difficulty paying for medical care. Neighborhood resources were assessed by asking questions related to physical activity resources located in the participant’s neighborhood, or within a 10–15 min walk from their home [16]. Detailed information on how each item was assessed is listed in Supplementary Table 1.

Each of the 12 items was dichotomized in which participants were assigned a score of 1 if adverse (e.g., unsafe neighborhood) or 0 otherwise (e.g., safe neighborhood). For binary items, adverse conditions were coded directly (e.g., lacking health insurance = 1, having health insurance = 0). For items assessed on ordinal or Likert scales, responses were aggregated into composite scores and then dichotomized based on the distribution within the sample. Specifically, composite scores for social support, neighborhood cohesion, and neighborhood physical environment were divided into tertiles, with the most adverse tertile coded as 1 and the remaining tertiles coded as 0. For continuous measures, cut points were informed by established socially meaningful thresholds where available — for example, household income was dichotomized at $50,000 (below = adverse), reflecting a commonly used threshold for economic hardship in population-based research. Full details of the survey items, response scales, and analytic recodes for each of the 12 items are provided in Supplementary Table 1. Among the 5 domains, some (e.g., community and social context) included significantly more items than others (e.g., education); therefore, we weighted the domains to ensure that they had equal weights, irrespective of the number of items included in each. Weighting was done such that each domain had a total possible score of 0.2. The scores of the 5 domains were then summed to define the final SDOH aggregate index, which ranged from 0 to 1, with higher scores representing more adverse SDOHs. The distribution of SDOH scores was then divided into quartile for the statistical analyses, with quartile 1 representing the least disadvantaged and quartile 4 the most disadvantaged.

### 2.3 Cognitive function assessment

CARDIA technicians who underwent formal training and certification administered a battery of cognitive tests at the years 25, 30, and 35 visits and included the Digit Symbol Substitution Test (DSST), the Stroop Test, and the Rey Auditory Verbal Learning Test (RAVLT) [3]. The three cognitive tests were selected as part of the standardized CARDIA cognitive battery, administered consistently across examinations, ensuring comparability across time points and consistency with prior CARDIA cognitive studies [17]. Beyond their inclusion in the CARDIA battery, each test captures distinct yet complementary cognitive domains particularly relevant to midlife brain aging and early dementia risk. The Digit Symbol Substitution Test (DSST), a subtest of the Wechsler Adult Intelligence Scale (3rd edition), assesses most prominently visual motor speed, sustained attention, and working memory, domains known to show early decline with aging and which have demonstrated robust associations with white matter integrity in prior CARDIA analyses [18]. The range of scores is 0 to 133, with higher scores indicating better performance. The Rey Auditory Verbal Learning Test (RAVLT) assesses the ability to memorize and to retrieve words (verbal memory), among the earliest cognitive domains affected in AD pathology and sensitive to hippocampal integrity [19]. Results from the long delay (10 min) free recall were used in analyses. The range of scores is 0 to 15, with higher scores indicating better performance**.** The Stroop Test evaluates the ability to view complex visual stimuli and to respond to one stimulus dimension while suppressing the response to another dimension, an “executive” skill largely attributed to frontal lobe function[18] and sensitive to the effects of vascular risk factors and socioeconomic stress in midlife populations [17,18]. The interference score provides a measure of how much additional executive processing is needed to respond to an incongruent trial; thus, a higher interference score indicates worse performance on the task. For ease of interpretation in relation to the DSST and RAVLT, we reversed the scale of the Stroop task whereby higher scores represent better performance.

### 2.4 Brain MRI measures

Starting at the year 25 visit, a subset of CARDIA participants completed MRI brain scanning at three of the four clinic sites (Birmingham, Alabama; Minneapolis, Minnesota; and Oakland, California) with approximate balance within four strata of race (Black, White) and sex (male/female) [20]. The MRI scans were acquired on 3T scanners located at each CARDIA study site: Siemens 3T Tim Trio/VB15 platform in Minneapolis and in Oakland, and Philips 3T Achieva/2.6.3.6 platform in Birmingham. Standard quality assurance protocols using phantoms previously developed for the Functional Bioinformatics Research Network and the Alzheimer’s Disease Neuroimaging Initiative were used. Structural images used for this study were acquired with 1 mm isotropic 3D T1 and T2 sequences. Scan acquisition parameters have been previously described [20], and were processed using previously described methods [[21], [22], [23]]. In brief, structural images were processed using an automated multispectral computer algorithm that classified all supratentorial brain tissue into gray matter, white matter, and cerebrospinal fluid, and identifies anatomic regions of interest (ROI). After correction of intensity inhomogeneities [24], a multi-atlas skull stripping algorithm was applied for the removal of extra-cerebral tissues [25]. Each T1-weighted scan was then automatically segmented into a set of anatomical ROIs using a multi-atlas label fusion method [26]. The images were visually checked for incidental findings, motion artifacts, and other quality issues. White matter was classified into regions of interest according to the Jakob atlas and further into normal and abnormal tissue.^20^ The WMH volume was estimated from the sagittal 3-dimensional FLAIR, T1, and T2 sequences that contain tissue damaged because of ischemia, demyelination or inflammation, and penumbra surrounding brain infarcts. Total intracranial volume was estimated from the sagittal 3-dimensional T1 sequence as a measure of head size. The unit of measurement for the MRI measures is cm^3^.

### 2.5 Covariates

Demographic characteristics and cigarette smoking (current or former) were based on self-report. Body mass index (BMI) was calculated as weight in kilograms divided by height in meters squared. Hypertension was defined as systolic BP ≥130 mmHg, diastolic BP ≥80 mmHg. Physical activity was measured with the CARDIA Physical Activity History questionnaire which queries the amount of time per week spent in 13 categories of leisure, occupational, and household physical activities over the past 12 months [27]. Physical activity level was summarized as units of total activity incorporating moderate and high intensity activities. Diabetes mellitus at baseline was defined as fasting plasma glucose ≥126 mg/dL, oral glucose tolerance test ≥200 mg/dL, glycosylated hemoglobin ≥6.5%, or use of diabetes medications. A digital blood pressure monitor (Omron HEM-907XL; Online Fitness) was used to measure systolic and diastolic blood pressure. Depressive symptoms were assessed using the center for Epidemiological Studies Depression scale [28].

### 2.6 Analytic sample

The SDOH components were assessed at either year 15 or 20 or both. We included the 4016 participants who had attended at least one of these visits. When the SDOH component was available for both years, an average was calculated. Further inclusion criteria required at least two cognitive assessments completed across the three study visits, resulting in an analytical cohort of 3488 participants. Of the 3488 participants, 262 had SDOH measures available at Year 15 only, 267 at Year 20 only, and 3135 at both visits. When data were available at both time points, values were averaged to provide a more stable and representative estimate of SDOH exposure during early midlife, as single time point measures may be subject to short-term fluctuation. The MRI sub-sample (*n* = 645) consisted of participants who had at least two MRI examination across the 10 years of follow up. Compared to the main sample at baseline, the participants of the MRI analytic sample were more likely to be White, have more years of education, and less likely to smoke (*p* < 0.001 for all).

### 2.7 Statistical analysis

Participant characteristics across SDOH quartiles were compared using the χ^2^ tests for categorical variables and one-way ANOVAs for continuous variables, with Bonferroni-adjusted pairwise comparisons.

We used linear mixed effects regression to examine the association between level and 10-year change in the cognitive measures and our primary predictor, the overall SDOH index as a continuous measure and as categories. The fixed effects of the model included the SDOH quartile, linear follow-up time (5-year interval, maximum 10 years in total), and their interaction term. Random effects included intercept and slope for time. Margins post-estimation was computed to calculate the adjusted mean difference in the cognitive tests at baseline among the groups. The mean difference in change over time was calculated as β_time_ + β_timexgroup_. The models were first adjusted for demographic factors (age, sex, and race) and then for vascular risk factors (hypertension, diabetes, physical exercise, BMI) and depressive symptoms, which have been shown to be associated with midlife cognition [3,29]. The same model paradigm was used for testing the association between the individual SDOH domains (as z-scores) and cognitive measures.

To test the association between SDOH quartiles and change in GMV and WMHs in the MRI sub-sample, we used linear mixed effects regression. Due to skewness, WMH was modeled as a natural logarithm of WMH. Coefficients from models with log-transformed WMH are presented as exponentiated ratios, representing the multiplicative change in WMH volume per unit increase in the predictor. Adjusted geometric mean WMH volumes at each time point are presented graphically. The fixed effects of the model included the SDOH quartile, linear follow-up time, and their interaction term. Random effects included intercept and slope for time. The models were first adjusted for demographic factors (age, sex, race, and education), intracranial volume, and scanning site and then for additional covariates (hypertension, diabetes, physical exercise, BMI, depression). The same model was used for testing the association between the individual SDOH domains (as z-scores) and MRI brain measures.

Moreover, we tested interactions between the SDOH index and sex and race respectively on the cognitive and brain outcomes.

All statistical analyses were performed using Stata SE 17.0 (StataCorp LP).

## 3 Results

At baseline (Year 20), participants’ mean age was 40 (range 32 to 55), 57% (*n* = 1981) were female, and 47% were Black (*n* = 1625). The distribution of the SDOH index was slightly left skewed with a median estimated at 0.17 (interquartile range 0.10–0.59). The participants in the highest quartile (most disadvantage) were more likely to be Black, current/former smoker, have diabetes, more likely to have hypertension, higher BMI, lower physical exercise and higher depressive symptoms (Table 1).

**Table 1:** Population characteristics by social determinants of health index quartile (*n* = 3488).

	SDOH Index				
Variable, mean (sd) or n (%)	Quartile 1 (least disadvantage)	Quartile 2	Quartile 3	Quartile 4 (most disadvantage)	Overall *p* value
Age	41.1 (3.6)	40.4 (3.8)	40.4 (3.8)	40.2 (4.0)	<0.001^a^^,^^b^^,^^c^
Female sex	566 (53.6)	458 (59.3)	465 (58.8)	493 (56.7)	0.056
Black race	262 (24.9)	367 (47.5)	434 (54.9)	562 (64.6)	<0.001^a^^,^^b^^,^^c^^,^^d^^,^^e^^,^^f^
Current/former smoker	322 (30.6)	261 (33.8)	326 (41.3)	465 (53.5)	<0.001^b^^,^^c^^,^^d^^,^^e^^,^^f^
Diabetes	51 (5.2)	46 (6.4)	57 (8.0)	86 (11.0)	<0.001^c^^,^^e^
Hypertension	172 (16.3)	157 (20.3)	185 (23.4)	215 (24.7)	<0.001^a^^,^^b^
Physical activity, exercise units	386.3 (294.2)	355.3 (275.9)	329.7 (268.8)	316.4 (268.5)	<0.001^b^^,^^c^^,^^d^
Body mass index, kg/m^2^	28.0 (6.4)	28.8 (6.2)	30.1 (7.2)	29.8 (7.1)	<0.001^b^^,^^c^^,^^d^^,^^e^
Depressive symptoms	6.2 (4.9)	8.6 (6.1)	10.2 (7.2)	12.5 (8.4)	<0.001^a^^,^^b^^,^^c^^,^^d^^,^^e^^,^^f^

Bonferroni-adjusted 0.05/6 = 0.0083 significant difference:.

a Q1 vs Q2;.

b Q1 vs Q3;.

c Q1 vs Q4;.

d Q2 vs Q3;.

e Q2 vs Q4;.

f Q3 vs Q4.

**Fig. 1:**
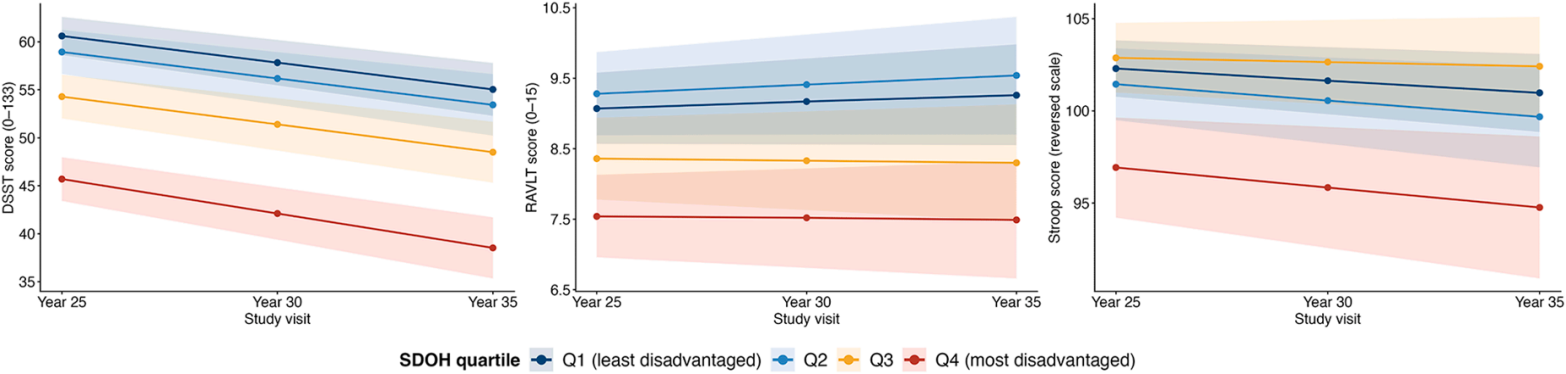
SDOH index quartile and level and change in cognitive measures over 10 years (*n* = 3488).

At baseline, the most disadvantaged group (Q4) performed worse on the DSST (adj. mean: 60.80, 95% CI: 59.88 to 61.72), RAVLT (adj. mean: 7.65, 95% CI: 7.47 to 7.84) and the Stroop (adj. mean: 101.49, 95% CI: 100.71 to 102.28) compared to the least disadvantaged group (Q1; DSST adj. mean: 72.36, 95% CI: 71.52 to 73.20; RAVLT adj. mean: 8.67, 95% CI: 8.50 to 8.84; Stroop adj. mean: 105.09, 95% CI: 104.53 to 105.65), *p* < 0.001 for all (Fig. 1). Baseline differences were also present for intermediate disadvantage quartile 3 in DSST (adj. mean: 65.67, 95% CI: 64.68 to 66.67) and the Stroop (adj. mean: 103.87, 95% CI: 103.23 to 104.51), *p* < 0.001 for all, and quartile 2 in DSST (adj. mean: 70.42, 95% CI: 69.42 to 71.43, *p* = 0.007), compared to the least disadvantaged group. The differences were attenuated after additional adjustment for vascular risk factors and depressive symptoms for those with intermediate disadvantage (quartile 2 and 3) but remained significant for the most disadvantaged quartile (DSST adj. mean: 62.73, 95% CI: 61.75 to 63.72; RAVLT adj. mean: 7.93, 95% CI: 7.73 to 8.13; Stroop adj. mean: 102.22, 95% CI: 101.40 to 103.03).

Figure legend: Adjusted mean cognitive test score trajectories across SDOH quartiles over 10 years of follow-up (Years 25–35), for (A) DSST, (B) RAVLT, and (C) Stroop. Q1 represents the least disadvantaged and Q4 the most disadvantaged group. Shaded bands represent 95% confidence intervals. Higher scores indicate better performance for DSST and RAVLT; the Stroop scale is reversed such that higher scores indicate better performance. Models are adjusted for age, sex, and race.

Over the follow up, the most disadvantaged participants had significantly steeper decline on the DSST (adj. mean change: −0.72, 95% CI: −0.93 to −0.52) compared to the least disadvantaged (adj. mean change: −0.55, 95% CI: −0.76 to −0.34, *p* = 0.013), but not on the RAVLT (Q4 adj. mean change: −0.01, 95% CI: −0.03 to 0.02; Q1 adj. mean change: 0.02, 95% CI: −0.005 to 0.041) or Stroop (Q4 adj. mean change: −0.22, 95% CI: −0.333 to −0.100; Q1 adj. mean change: −0.13, 95% CI: −0.197 to −0.070), with no significant difference in rate of decline compared to Q1 (*p* = 0.679 and *p* = 0.208, respectively). Results were similar after additional adjustment for vascular risk factors and depressive symptoms. The most disadvantaged group continued to show steeper decline on the DSST (Q4 adj. mean change: −0.71, 95% CI: −0.81 to −0.61; Q1 adj. mean change: −0.55, 95% CI: −0.63 to −0.46, *p* = 0.016). There was no significant differential decline in RAVLT (Q4 adj. mean change: −0.00, 95% CI: −0.03 to 0.02, *p* = 0.856) or Stroop (Q4 adj. mean change: −0.22, 95% CI: −0.338 to −0.101, *p* = 0.208) by SDOH quartile.

**Fig. 2:**
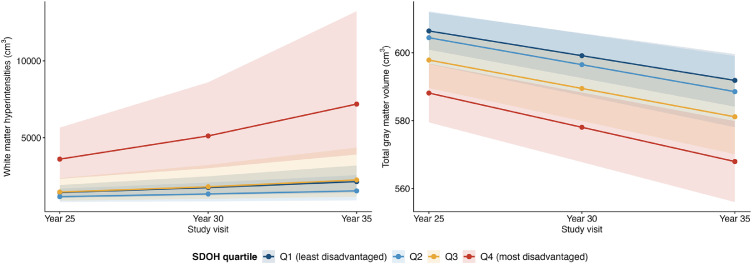
SDOH index quartile and level and change in brain measures (cm^3^) over 10 years (*n* = 645).

Compared to the overall sample, the MRI sub-sample were less likely to be Black and were more likely to belong to the least disadvantaged group (*p* < 0.05). At baseline, the most disadvantaged group had WMH volume 1.19 times higher than the least disadvantaged group (ratio: 1.19, 95% CI: 0.94 to 1.49, *p* = 0.149), though this did not reach statistical significance. Over the follow-up period, the most disadvantaged group showed significantly faster WMH accumulation compared to the least disadvantaged group, with WMH volume increasing by a factor of 1.07 (95% CI: 1.05 to 1.09) per 5-year interval in Q4 compared to 1.04 (95% CI: 1.03 to 1.05) in Q1 (*p* = 0.007). Adjusted geometric mean WMH volumes at each time point are shown in Fig. 2. For total GM, the most disadvantaged group (Q4) had a lower adjusted mean total GM volume compared to the least disadvantaged group (Q1; 628.80 cm³, 95% CI: 624.28 to 633.33 vs. 635.74 cm³, 95% CI: 632.59 to 638.88), though this difference did not reach statistical significance (*p* = 0.172). The most disadvantaged group showed a steeper decline in total GM volume compared to the least disadvantaged group (−2.02 cm³, 95% CI: −2.39 to −1.65 vs. −1.46 cm³, 95% CI: −1.71 to −1.20 per 5-year interval, *p* = 0.011) (Fig. 2). After additional adjustment for vascular risk factors and depressive symptoms, the most disadvantaged group continued to show significantly greater WMH accumulation (Q4 ratio: 1.07, 95% CI: 1.05 to 1.09; Q1 ratio: 1.04, 95% CI: 1.03 to 1.05, *p* = 0.007) and a steeper decline in total GM volume (Q4: −1.91 cm³, 95% CI: −2.29 to −1.53; Q1: −1.43 cm³, 95% CI: −1.69 to −1.18, *p* = 0.011) per 5-year interval compared to the least disadvantaged group.

Figure legend: Adjusted mean brain measure trajectories across SDOH quartiles over 10 years of follow-up (Years 25–35), for (A) white matter hyperintensity volume and (B) total gray matter volume. Q1 represents the least disadvantaged and Q4 the most disadvantaged group. Shaded bands represent 95% confidence intervals. White matter hyperintensity volume is back-transformed from log scale and presented in cm³. Total gray matter volume is presented in cm³. Models are adjusted for age, sex, race, intracranial volume, and scanning site.

For the separate SDOH domain-specific models, lower economic stability and worse health care access and lower education were associated with lower scores on all cognitive tests. Social support was associated with lower baseline DSST scores but not RAVLT or Stroop. Neighborhood context was not significantly associated with any cognitive measure at baseline. Over time, worse healthcare access was associated with accelerated decline in Stroop performance, while lower economic stability was associated with accelerated decline in DSST. None of the domains were significantly associated with accelerated decline in RAVLT, and neighborhood context was not associated with change in any cognitive measure over time (Table 2).

**Table 2:** Baseline adjusted mean difference and change over time in cognitive measures by individual social determinants of health domains (*n* = 3488).

Domain	DSST	RAVLT	Stroop
SDOH index	−4.72 (−5.23 to −4.21)*	−0.43 (−0.53 to −0.32)*	−1.62 (−2.03 to −1.21)*
SDOH index x time	−0.05 (−0.10 to 0.00)	−0.01 (−0.02 to 0.00)	−0.02 (−0.08 to 0.03)
Economic stability	−3.84 (−4.35 to −3.34)*	−0.29 (−0.40 to −0.19)*	−0.97 (−1.34 to −0.61)*
Economic stability x time	−0.04 (−0.08 to 0.01)	−0.01 (−0.03 to 0.02)	−0.04 (−0.08 to 0.01)
Health care access	−3.29 (−3.78 to −2.80)*	−0.22 (−0.32 to −0.12)*	−1.35 (−1.71 to −1.00)*
Health care access x time	−0.03 (−0.08 to 0.02)	−0.01 (−0.03 to 0.02)	−0.03 (−0.08 to −0.02)*
Social support	−1.07 (−1.59 to −0.55)*	0.05 (−0.06 to 0.16)	0.10 (−0.27 to 0.47)
Social support x time	−0.04 (−0.09 to 0.01)	−0.00 (−0.03 to 0.02)	−0.02 (−0.07 to 0.02)
Neighborhood context	−0.14 (−0.69 to 0.41)	−0.00 (−0.11 to 0.11)	−0.22 (−0.60 to 0.16)
Neighborhood context x time	−0.03 (−0.08 to 0.02)	0.02 (−0.01 to 0.04)	−0.02 (−0.02 to 0.07)
Education	−3.96 (−4.44 to −3.48)*	−0.53 (−0.63 to −0.43)*	−1.63 (−1.98 to −1.28)*
Education x time	−0.01 (−0.06 to 0.04)	0.00 (−0.02 to 0.03)	−0.01 (−0.05 to 0.03)

Abbreviations: DSST, digit symbol substitution test; RAVLT, Rey auditory verbal learning test.

Estimates represent differences per 1 SD increase in each SDOH domain z-score and per 1 SD increase in the overall SDOH index. Baseline estimates reflect cross-sectional associations at Year 25; change estimates reflect the interaction between each SDOH measure and follow-up time, representing the additional rate of change per year per 1 SD worse score.

⁎ *p* < 0.05.

For the brain outcomes, lower education was associated with baseline higher WMHs and worse health care access was associated with faster accumulation of WMHs. For total GM, lower economic stability, worse health care access, and education were significantly associated with a steeper decline. When modeled continuously, the overall SDOH index showed a borderline association with higher WMH at baseline and a significant association with steeper GM decline over time (Table 3).

**Table 3:** Baseline adjusted mean difference and change over time in brain measures by individual social determinants of health domains (*n* = 645).

Domain	WMHs	Total GM volume
SDOH index	1.08 (0.99 to 1.17)	−1.26 (−3.51 to 1.00)
SDOH index x time	1.00 (1.00 to 1.02)	−0.20 (−0.34 to −0.04)*
Economic stability	0.98 (0.88 to 1.07)	−2.30 (−4.88 to 0.10)
Economic stability x time	1.01 (1.00 to 1.02)	−0.19 (−0.35 to −0.04)*
Health care access	1.06 (0.97 to 1.16)	−1.00 (−3.45 to 1.46)
Health care access x time	1.01 (1.00 to 1.02)*	−0.17 (−0.33 to −0.01)*
Social support	0.98 (0.89 to 1.08)	−1.15 (−3.50 to 1.19)
Social support x time	1.01 (1.00 to 1.01)	−0.04 (−0.19 to 0.10)
Neighborhood context	1.07 (0.98 to 1.16)	0.63 (−1.82 to 3.09)
Neighborhood context x time	1.00 (0.99 to 1.01)	−0.00 (−0.15 to 0.15)
Education	1.09 (1.00 to 1.19)*	−0.34 (−2.77 to 2.09)
Education x time	1.01 (1.00 to 1.02)	−0.18 (−0.34 to −0.02)*

Abbreviations: WMHs, white matter hyperintensities; GM, gray matter.

All models adjusted for age, sex, race, and intracranial volume. SDOH domain scores are expressed as z-scores. For white matter hyperintensities, ratios represent the change in WMH volume per 1 SD worse score on each domain; ratios below 1 indicate lower WMH volume and ratios above 1 indicate higher WMH volume. For total gray matter volume, coefficients represent the absolute difference in cm³ per 1 SD worse score on each domain. Baseline estimates reflect cross-sectional associations at Year 25; change estimates reflect the interaction between each domain and follow-up time, representing the additional rate of change per year per 1 SD worse domain score.

⁎ *p* < 0.05.

There were no significant interactions between the SDOH index and race and sex respectively on the association with the individual cognitive and brain outcomes (*p* > 0.10)

## 4 DISCUSSION

In this study of community-dwelling adults, we developed a SDOH index with components of economic stability, neighborhood and physical environment, education, community and social context, and health care access and quality, and examined the relationship between the index in early midlife and change in cognition and brain outcomes over ten years of follow up. At baseline, we saw stark differences in performance across all three cognitive measures between those with the highest disadvantage and the least disadvantage. Our findings indicated that higher disadvantage was associated with accelerated rate of decline in our measure of working memory, motor speed, and sustained attention (DSST). Higher disadvantage was also associated with greater accumulation of WMH and accelerated decline of GM compared to the lowest disadvantage. Altogether, these findings suggest that high social disadvantage is linked to large differences and worse brain integrity – and that the cognitive effects of disadvantage in early midlife can already be seen later in midlife.

The differential association between social disadvantage and decline specifically on the DSST, but not the RAVLT or Stroop, warrants consideration. The DSST is a particularly sensitive marker of early cognitive aging, as it integrates multiple cognitive processes — processing speed, sustained attention, and working memory — and relies heavily on white matter integrity and frontal-subcortical circuitry, which are among the earliest structures affected by vascular and social risk factors [17,18]. In contrast, the RAVLT, which measures verbal episodic memory, and the Stroop, which measures executive function, may reflect cognitive domains that are more resistant to change over the relatively short 10-year follow-up window in midlife, or may require longer follow-up to detect differential decline by social disadvantage. It is also possible that the DSST, as a timed test of psychomotor speed, is more sensitive to the cumulative physiological effects of chronic stress — such as vascular burden, inflammation, and dysregulated cortisol — which have been disproportionately linked to social disadvantage and are known to particularly affect processing speed [17,30]. Additionally, the baseline differences already observed across all three cognitive measures suggest a potential floor effect for the RAVLT and Stroop in the most disadvantaged group, which may have limited the ability to detect differential rates of decline over time on these measures.

Our study is among the first to utilize a comprehensive SDOH index to examine the relationship between SDOH and cognitive aging and brain health in midlife. Previous studies on cognitive aging have focused largely on individual risk factors of SDOH in older adults. For instance, one study found that aspects of SDOH such as perceived greenspace access and time spent in greenspaces were associated with resilience against dementia, and interpersonal discrimination was associated with vulnerability to dementia [31]. There have also been studies of smaller composites of SDOH factors in older adults, showing a similar link between disadvantage within these SDOH factors and greater cognitive decline or odds of cognitive impairment [2,30,[32], [33], [34], [35]]. For example, the Area Deprivation Index (ADI)[36] is one such composite measure linked to higher risk of dementia [33], while it integrates several SDOH factors like income, education, employment, and housing to examine neighborhood disadvantage, further domains such as social/community context and health care access and quality are not included. Another study in older adults found that accumulation of unfavorable SDOH is linked to decreased cognitive performance [2]. In our study, the inclusion of both the aggregate SDOH measure and additional analysis by individual domains revealed a point of interest: while there was some overlap, the individual domains showed differential associations with cognitive and brain measures at baseline and over time, indicating that an aggregate measure including all SDOH domains provides a more comprehensive view of disparities associated with social disadvantage. Notably, economic stability, education, and healthcare access emerged as the most consistently associated domains across cognitive and brain outcomes, while social support showed a selective association with baseline processing speed but not with decline over time and neighborhood context showed little association with any outcome. This pattern may reflect the particular salience of material resources and human capital for brain health in midlife, though the null findings for other domains may reflect limited power or context-specific factors rather than true absence of effect.

Within the limited research of SDOH and cognitive outcomes, there is notably scarce research on middle-aged adults. Our sample from the CARDIA cohort was in early midlife at baseline, then followed for 10 years during the transition through midlife into old age, allowing us not only to examine the impact of an aggregate SDOH index in midlife, but also to capture how SDOH differentiates the beginning stages of aging. Notably, the most disadvantaged already held baseline differences in cognitive measures and total GM– indicating that social disadvantage was likely exerting influence on these outcomes prior to baseline (mean age 40). Additionally, WMHs are typically subtle and less prevalent in early midlife compared to older populations, however, our findings demonstrate that differential accumulation across SDOH quartiles is already detectable in this age group. This highlights the importance of studying brain aging trajectories before pathological changes become more overt, and suggests that midlife may represent a critical window for identifying early neurobiological signatures of social disadvantage. In addition, the attenuation of associations when modeling the SDOH index continuously suggests a threshold effect whereby cognitive impacts of social disadvantage may be concentrated in the most disadvantaged group.

Several mechanistic pathways between social adversity and brain health have been proposed. For instance, chronic stressors tied to social and environmental contexts, such as discrimination or neighborhood violence, have been associated with physiological changes (e.g., elevated cortisol levels) that impact cognitive function [37,38]. Studies in younger populations, highlight how adverse childhood experiences and environmental stressors can negatively affect brain development and cognitive trajectories, which would potentially explain why we found differences already at midlife in the most disadvantaged group [39]. The domain-specific analyses further illuminate these pathways: lower economic stability may increase chronic stress and poorer health behaviors [30], worse healthcare access may reflect delayed management of vascular risk factors such as hypertension and diabetes [40], and the more specific association of lower education with verbal memory may reflect cognitive reserve mechanisms, while economic and healthcare-related stressors more broadly affect processing speed and executive function through vascular burden [18]. These mechanisms remain speculative, but align with the recent Lancet Commission on dementia prevention, which estimated that up to 45% of dementia cases may be preventable through risk factor modification [41]. Together, these findings suggest that addressing upstream social determinants may complement individual-level risk factor interventions in reducing the population burden of dementia.

Our study has several strengths, including its population-based design and large cohort of adults in midlife, as well as our creation and use of a comprehensive SDOH measure drawing from all five domains of SDOH. In addition, CARDIA provides a sample of participants with 10-year follow up in cognitive and MRI data, allowing us to examine longitudinal changes in both cognitive measures and structural brain health across a decade. Limitations of the study include lack of diversity among the CARDIA participants, as the cohort was comprised of only Black and White adults, as well as within the MRI subsample, which was more likely to be White, higher educated, and in better health than the rest of the cohort. As a result, our findings may not be entirely generalizable to other racial and ethnic groups. Additionally, participants were recruited from four U.S. cities, limiting generalizability to other contexts. In the United States, healthcare access is strongly tied to economic resources, which may amplify observed associations; in settings where healthcare is more universally guaranteed, these relationships may differ. Moreover, while the aggregate SDOH index provides a comprehensive summary of cumulative social disadvantage, the assumption of equal domain weighting may obscure heterogeneity in how individual indicators relate to brain health across subgroups defined by race, sex, or age. Future studies should consider more granular approaches, such as profile analysis or longitudinal structural equation modeling, to examine how distinct patterns of SDOH exposure relate to cognitive and brain aging trajectories. Finally, We tested for effect modification by sex and race, and no significant interactions were detected. However, given the well-documented disparities in both SDOH exposure and cognitive aging across sex and racial groups, it is possible that our study was underpowered to detect meaningful differences in the association between SDOH and brain health outcomes by these subgroups. Future studies with larger and more diverse samples are needed to examine whether the effects of social disadvantage on brain aging differ by sex and race.

This study utilizes a comprehensive SDOH measure to provide insight regarding social disadvantage’s relationship to brain aging at midlife, indicating that high social disadvantage is associated with accelerated cognitive decline and worsened brain integrity. These findings emphasize the need for prevention strategies and intervention efforts that target SDOH by midlife or earlier. Such strategies may include policies aimed at reducing economic instability through income support and affordable housing, improving healthcare access through community-based care models and insurance expansion, and investing in neighborhood-level infrastructure to reduce chronic stress burden. Given that cognitive differences were already present at mean age 40 in the most disadvantaged group, these efforts may need to begin well before midlife to meaningfully alter brain health trajectories.

## Declaration of the use of generative AI and AI-assisted technology

No generative AI or AI-assisted technology was used in the scientific writing, images, or artwork in this manuscript.

## Funding

The Coronary Artery Risk Development in Young Adults Study (CARDIA) is supported by contracts 75N92023D00002, 75N92023D00003, 75N92023D00004, 75N92023D00005, and 75N92023D00006 from the National Heart, Lung, and Blood Institute (NHLBI). CARDIA was also partially supported by the Intramural Research Program of the National Institute on Aging (NIA) and an intra-agency agreement between 10.13039/100010935NIA and NHLBI (AG0005). The CARDIA Cognitive Function ancillary study is supported by the National Institute on Aging (NIA) R01AG063887 and NHLBI R01HL122658 (multiple principal investigators: K. Yaffe and S. Sidney). This work was also supported by 10.13039/100021901NIA R35AG071916 (Yaffe).

## Ethical statement

At each examination, participants provided written informed consent, and study protocols were approved by institutional review boards at each study site and the CARDIA Coordinating Center.

## Data sharing

CARDIA data are available on reasonable request from the CARDIA Coordinating Center. CARDIA investigators are eager to collaborate with investigators interested in using CARDIA data. Please see the CARDIA website (cardia.dopm.uab.edu) for publications policies and for a list of CARDIA investigators. CARDIA data are also publicly available on the NIH-supported BioLINCC and dbGaP platforms.

## CRediT authorship contribution statement

**Christina S. Dintica:** Writing – review & editing, Writing – original draft, Project administration, Methodology, Investigation, Formal analysis, Data curation, Conceptualization. **Julia Cheunkarndee:** Writing – review & editing. **R. Nick Bryan:** Data curation. **Lenore J. Launer:** Data curation. **Kristine Yaffe:** Writing – review & editing, Supervision, Data curation.

## Declaration of competing interest

The authors declare the following financial interests/personal relationships which may be considered as potential competing interests:

Kristine Yaffe reports financial support was provided by 10.13039/100000049National Institute on Aging. If there are other authors, they declare that they have no known competing financial interests or personal relationships that could have appeared to influence the work reported in this paper.

## Appendix Supplementary materials

Image, application 1
